# Evaluation of a passive wearable arm ExoNET

**DOI:** 10.3389/frobt.2024.1387177

**Published:** 2024-07-10

**Authors:** Partha Ryali, Valentino Wilson, Courtney Celian, Adith V. Srivatsa, Yaseen Ghani, Jeremy Lentz, James Patton

**Affiliations:** ^1^ Neuro-Machine Interaction Lab, Department of Biomedical Engineering, University of Illinois at Chicago, Chicago, IL, United States; ^2^ Robotics Lab, Center for Neuroplasticity, Shirley Ryan AbilityLab, Chicago, IL, United States

**Keywords:** medical Robot, wearable Design, Exoskeletons, pilot Study, gravity compensation

## Abstract

Wearable ExoNETs offer a novel, wearable solution to support and facilitate upper extremity gravity compensation in healthy, unimpaired individuals. In this study, we investigated the safety and feasibility of gravity compensating ExoNETs on 10 healthy, unimpaired individuals across a series of tasks, including activities of daily living and resistance exercises. The direct muscle activity and kinematic effects of gravity compensation were compared to a sham control and no device control. Mixed effects analysis revealed significant reductions in muscle activity at the biceps, triceps and medial deltoids with effect sizes of −3.6%, −4.5%, and −7.2% rmsMVC, respectively, during gravity support. There were no significant changes in movement kinematics as evidenced by minimal change in coverage metrics at the wrist. These findings reveal the potential for the ExoNET to serve as an alternative to existing bulky and encumbering devices in post-stroke rehabilitation settings and pave the way for future clinical trials.

## Introduction

Exoskeletons have long served as versatile tools to assist individuals, facilitate training, and offer therapy. By offsetting or entirely counteracting gravity’s effects, these devices simplify upper limb exercises, minimizing the joint torques required for arm movements. Evidence shows that such systems reduce muscle activation levels necessary for tasks like reaching, especially in muscles counteracting gravity (Coscia et al., 2014; Elias et al., 2016; Kristiansen et al., 2019). As the level of weight compensation increases, there is an observable enhancement in motion range and a decrease in muscle activity–factors associated with improving flexor synergies post-stroke. Passive exoskeletons may seem trivial and inefficacious, but their simplicity and low encumbrance make them a non-intimidating and attractive option for adoption in clinical settings (Sulzer et al., 2005; Gijbels et al., 2011).

Exoskeletons targeted for upper extremity rehab have been found to successfully deliver gravity assistance and facilitate recovery, but available devices are lacking. The Therapy Wilmington Robotic exoskeleton (T-WREX) (Housman et al., 2007) and its predecessor, The Armeo Spring, (Gijbels et al., 2011), are bulky, difficult to use, and require a steep learning curve to master proficiency. Popular actuated exoskeletons put limitations on clinical applications and uptake because users can be restricted to gamified VR environments instead of real-world tasks. The Armeo Spring and Myopro use continuous feedback from sensors, and the passive Saebo MAS (Fulton et al., 2020), Jaeco Arm Support (Atkins et al., 2008), and the Kuka Herder support (Herder et al., 2006) have single elastic elements crossing single joints, constraining the number of elastic elements. Also, some are not wearable, which may hinder clinical adoption.

**FIGURE 1:**
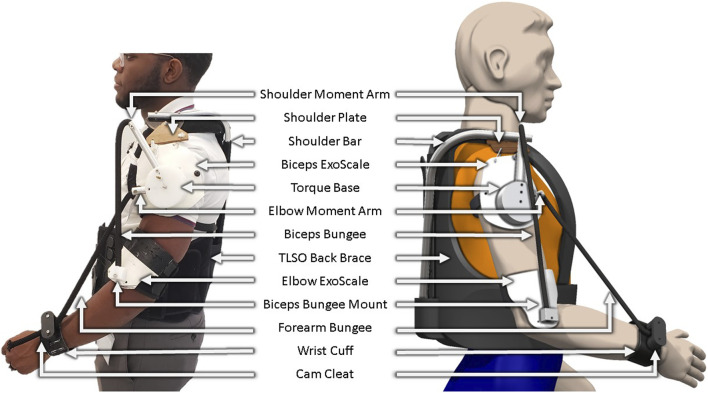
Wearable prototype. Physical device. CAD model with labelled components.

In prior work, we introduced a theoretical framework for ExoNETs (Exoskeletal Networks of Elastic Torque), a passive, simple, customizable, and wearable device designed to facilitate upper extremity rehabilitation. The ExoNET leverages stacks of spring elements which are optimized to approximate nonlinear, multijoint torque fields in both assistive and therapeutic applications in the parasagittal plane (Malizia et al., 2022; Malizia et al., 2020; Ryali et al., 2022; Ryali et al., 2020). Specifically, the ExoNET optimizes the multiple lines of action of passive linear elements used in combination. Each element acts as near sinusoidal basis function that can be summed with others to approximate an infinite set of possible torque-angle relationships with a network of springs. These simple springs can be single or multi-joint and may take advantage of their nonlinear properties such as stiffening and slacking. In prior work, successful network configurations have been identified for a range of assistive and rehabilitative applications such as gravity compensation, attractor fields, limit push fields (Shah et al., 2018), and error augmentation (Abdollahi et al., 2014; Malizia et al., 2022; 2020; Ryali et al., 2022; 2020). As the complexity of the desired torque profile increases, so does the size and complexity of the spring network needed to deliver that torque. However, we found gravity compensation to be one of the simplest, and here we focus on a multi-joint gravity-assist version (Figure 1) and evaluate its effects.

Studying safety and feasibility provides a vital first step in understanding the ability of any intervention, and testing on healthy adults is critical prior to clinical application. Several such studies (Xiloyannis et al., 2019; Proietti et al., 2021) have investigated anti-gravity effects by observing electromyographic (EMG) and spectral aspects of range of motion during specific movements. They also compared powered to unpowered control conditions.

Here, we similarly continue our work by testing the safety and feasibility of an anti-gravity implementation of our passive wearable ExoNET. While this is not the first passive solution for upper extremity gravity compensation (Chheta et al., 2017; Asgari and Crouch, 2021; Asgari et al., 2022), to our knowledge this is the first instance of a personalized, wearable, passive solution that enables full range of motion. We asked 10 unimpaired adults to perform a series of activities of daily living and resistance exercises to determine amounts of effort relief and motion encumbrance. We study the effects of anti-gravity compared to two control conditions: a *sham* where subjects wore the device with no forces engaged, and *without device*.

## Materials and methods

### Prototype design

We adapted our previous prototype (Ryali et al., 2022; Ryali et al., 2020) to emphasize features paramount to human use (Gull et al., 2020): lightweight construction, safety, comfort, and adaptability to different elastic network configurations. A unique feature introduced in this design is the *ExoScale*. Drawing inspiration from medieval armor, insect exoskeletons, and fish scales; ExoScales serve as wearable plates, which we designed out of formable thermoplastic. The upper extremity ExoNET incorporates two ExoScales: one positioned laterally to the glenohumeral joint, and one positioned laterally to the elbow joint. These ExoScales can be fabricated to different lengths which may enable them to slide or move underneath each other, maximizing the range of motion experienced by users.

The structural foundation of the ExoNET was a Thoracic Lumbar Sacral Orthosis (TLSO) back brace. An aluminum tube was affixed to the brace to support shoulder load. A shoulder plate allowed for 3D motion with a strap hinge connecting to the shoulder ExoScale. This plate is comprised of a semicircular shoulder board and a rotating platform. The board was anchored to the aluminum tube, while the rotating platform was screwed into the board on one end and attached to the ExoScale on the other end with strings that behaved like a hinge. This mechanism enabled users’ natural shoulder movements for flexion, extension, and abduction. A TLSO back brace was chosen as the foundation for the rest of the ExoNET design because of its ability to provide rigid support to the spine. With custom built backpacks and straps, ExoScales quickly moved out of alignment when they were under high spring loads. With the chosen TLSO back brace, we were able to comfortably and quickly fit the ExoNET to a wide range of body types because it can be prebuilt with a wide variety of straps and adjustment mechanisms.

The shoulder ExoScale was loosely affixed to the arm by a strap that helped maintain position over the shoulder joint center of rotation during parasagittal movements. A loose connection was required to ensure that the plate did not move with the arm during movements like shoulder abduction or flexion which would change the moment arm locations of the springs. An acrylic torque base was attached to the shoulder ExoScale and had holes to attach aluminum rotators too. These rotators served as origin points for the two bungee cords in this study. The two attachment points for the bungee cords were on the elbow ExoScale and wrist cuff (Figure 1). The torque base and rotators were designed with adjustability in mind and can be oriented differently to accommodate for different ExoNET configurations. ExoScales represent the layer of the device that directly interfaces with a user’s arms and serves as an important foundation for single or multiple stacks of springs.

For its current target application of gravity compensation, the aluminum rotators were fixed at a specific moment arm relative to the shoulder joint mechanism. However, for target applications that go beyond simple gravity assistance, the torque base and rotators can be easily re-orientated to another moment arm by adding in a longer (or shorter) rotator or changing the angle where the spring is attached to.

### Study methods

This study was a cross-over, randomized, single-blinded pilot study; testing the safety, muscle activity, and kinematic effects of the ExoNET prototype on 10 unimpaired, neurologically intact subjects.

### Participants

Ten healthy, unimpaired individuals (5 female and 5 male, age range 18–68, mean age 33.7±14.6) were invited to participate in this study. Participants were recruited through word of mouth and recruitment flyers posted around the Shirley Ryan AbilityLab. All participants were consented to before data collection began using an approved protocol by the Northwestern University (IRB Approval Number: STU00216062) and reliance with the University of Illinois at Chicago research ethics authority. This work conforms to the Declaration of Helsinki for research involving human subjects.

### Protocol

Understanding the direct muscle activity and kinematic effects of a gravity support ExoNET, along with its general safety and comfort were important factors in determining whether the ExoNET was feasible for future clinical use. To answer these questions, we conducted a preliminary, single-blind study on 10 healthy, unimpaired participants, evaluating the direct muscle activity and kinematic effects of a gravity support ExoNET across a series of 10 tasks, organized into 4 high-level task categories. The 4 high level categories and their corresponding tasks include: 5 Activities of Daily Living (Drinking Water, Combing Hair, Vertical Pick and Place Box, Horizontal Pick and Place Box, and Wipe Down a Whiteboard), 2 Range of Motion Tasks (unweighted bicep curl and shoulder circles), 2 Resistance Exercises with a 5 pound weight (bicep curl and shoulder raise), and a timed Free Exploration Task. For the timed free exploration task, participants were instructed to continuously move their arms for 6 min, with the goal of having their arms cover the entire range of motion across their reachable workspace. Besides the 6 min of free exploration, all participants performed 10 repetitions of each task. Activities of Daily Living (ADLs) were chosen in collaboration with a Shirley Ryan AbilityLab Occupational Therapist, and our goal was to have participants perform tasks across the entire range of their workspace.

Muscle activity and kinematic effects of the gravity support ExoNET were compared to two controls. The first control was a sham where participants wore an ExoNET with slack springs that delivered zero torque to the arms. Participants were not given explicit feedback about the springs being slack. In the second control, participants performed all tasks without the device. To mitigate the effects of practice and adaptation on subject muscle activities and kinematics, the first 5 subjects performed all tasks starting with the no device condition and ending with the gravity compensation condition last, while the second 5 subjects started with the gravity compensation condition and ended with the no device condition.

### Data analysis

Muscle activity effects were determined based on electromyography data (Bawa and Banitsas, 2022) (using the Trigno Delsys System) collected at the long heads of the biceps, triceps, and medial deltoid muscles. The primary muscle activity outcome was the effect of gravity compensation on the amplitude of muscle activation at the long heads of the biceps. Secondary muscle activity measures included muscle activation at the triceps and medial deltoid muscles. EMG signals were acquired at 2 khz, band-pass filtered (20–400 Hz), rectified, low-pass filtered (with a cut-off frequency of 5 Hz), and then integrated over 100-m intervals to obtain the EMG envelope time series. To normalize EMG amplitudes across subjects, it was important to collect information about each subject’s maximum voluntary contraction (MVC). MVC refers to the maximal force exerted by muscles during a single task or effort. To collect MVC information, we instructed participants to squeeze a stress ball while flexing all of their upper extremity muscles to their maximal capability for 20 s. The highest values attained during this test defined the MVC that EMG data was normalized to. We then estimated the root mean square value expressed as a percentage of MVC, RMSemg. We then formed distributions of RMSemg, and data was transformed if the Shapiro-Wilk test detected non-normality. Mixed effects methods evaluated differences between the no device and slack conditions to the gravity condition. Mixed effects considered the direct effects of device condition and random effects of task and subject (alpha = 0.05).

Kinematic effects were determined based on joint-tracking data collected with the Xbox Kinect hardware and Body2Basics (Mangal and Tiwari, 2020) software. Body2Basics provides a 3D position estimate for all joint positions in the upper and lower extremity. To mitigate the effects of inaccurate joint locations and noise, we removed the top 5% of outlier points that were furthest from the median of the point cloud distribution. Volume was calculated using the convex hull algorithm (Chadnov and Skvortsov, 2004), which fits the 3D space covered by the wrist into the shape of a polygon, which yielded *coverage*–a metric describing range of motion tendencies that might change when wearing the ExoNET. Coverage changes give us insight into movement capabilities and serve as a useful metric to understand how kinematics can change across tasks and conditions. Mixed effects analysis on group coverage metrics considered the direct effects of device condition and random effects of task and subject (alpha = 0.05).

## Results

### Gravity compensation showed reduced effort

**FIGURE 2:**
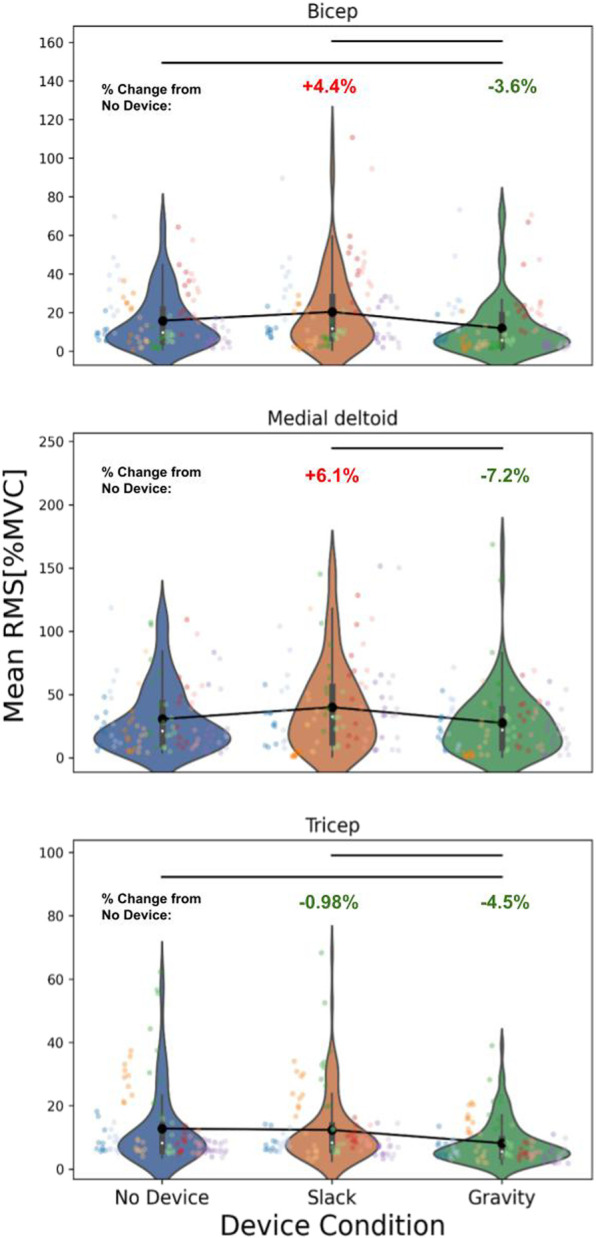
Overall muscle activity Trends in 10 Healthy, Unimpaired Participants. Individual Colors represent activity from individual subjects, with each dot representing the mean RMS [%MVC] for a specific task. Mean trend is plotted in black. Muscle activity significantly decreased between the no device and gravity condition for the biceps (effect size = −3.6, *p* < .0001), triceps (effect size = −4.5, *p* < .0001), and medial deltoid (effect size = −7.2, *p* < .0001), significantly increased between no device and slack for the biceps (effect size = 4.4, *p* < .0001) and medial deltoid (effect size = 6.1, *p* < .0001), and significantly decreased between no device and slack for the triceps (effect size = −.98, *p* < .0001). Units for all effect sizes is mean rms [%mvc].

Gravity compensation indeed reduced the amplitude of muscle activity across all 3 muscles (Figure 2) with an effect size of −3.6% MVC (*p* < .001) for the biceps, −4.5% MVC (*p* < .001) for the triceps, and −7.2% MVC (*p* < .001) for the medial deltoid. When participants performed tasks in the slack condition, they experienced a significant increase in muscle activity at the biceps (effect size = 4.4, *p* < .001) and medial deltoid (effect size = 6.1, *p* < .001) and a significant decrease in muscle activity at the triceps (effect size −0.98, *p* < .001).

**FIGURE 3:**
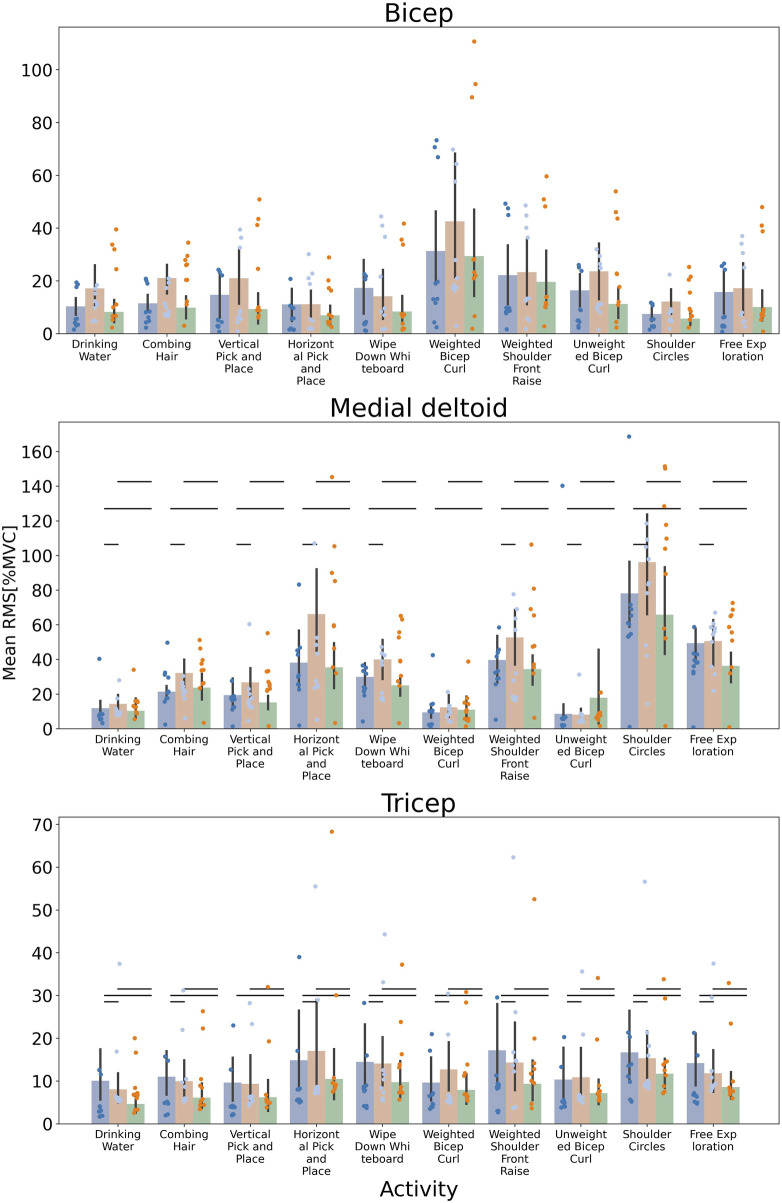
Muscle Activation Trends for Individual Tasks Across 10 Healthy Subjects The mean RMS [%MVC] is plotted for each task for the no device (blue), slack (orange), and gravity compensation (green) condition. Individual trends where biceps and triceps were the prime movers, such as bicep curls, resulted in the most dramatic decrease activity when gravity compensation was engaged. Conversely, non-parasagittal tasks such as shoulder circles led to increased shoulder muscle activity during gravity compensation. Non-parametric, Mann-Whitney significance tests were conducted for all pairwise combinations of activities. Significant differences are shown with horizontal bars.

While these overall trends revealed significant reductions in activity due to gravity compensation, we also observed significant *increases* due to the sham (slack) condition. Non-parametric significance tests revealed variable trends for all pairwise combinations of activities across the 3 muscles (Figure 3). For example, individual tasks focused primarily on the biceps, e.g., bicep curls, displayed greater decreases in activity compared to tasks such as free exploration. In non-parasagittal plane movements, there were increases in shoulder muscle activities, revealing limitations in the device’s gravity compensation capabilities.

### Ranges of motion were not altered by the device

**FIGURE 4:**
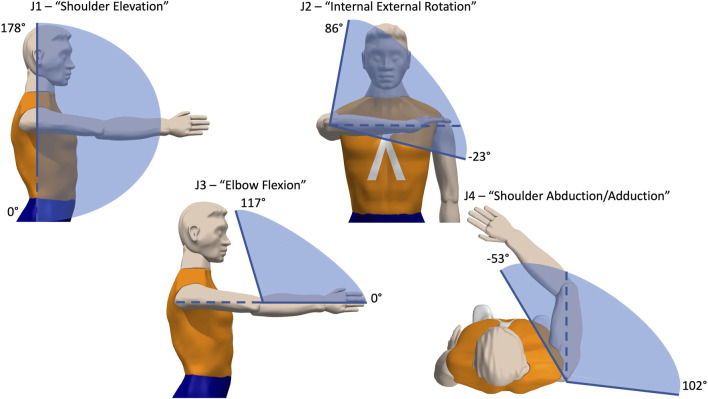
Resulting Joint Angle Limits for Wearable ExoNET Prototype. Four joint angle ranges are calculated relative to the reference angle drawn with a dotted line. These angle ranges show the inner 95th percentile of the movement distribution of wrist positions during a free exploration task. These were close to normal healthy human ranges (Aizawa et al., 2013). However, these functional ranges are for each joint in isolation, and should be measured further using multivariate statistics, as shown below.

In contrast to effort (muscle activity) measures, the range of motion of 4 joint angles demonstrated nearly full range of motion at the shoulder and elbow joints (Figure 4). This evaluated possible maximal range movements in the shoulder flexion, shoulder adduction/abduction, elbow flexion, and internal/external rotation. Overall, we observed highly capable ranges for each joint that were similar to joint angle ranges found in healthy adults (Aizawa et al., 2013), so while the functional encumbrance may remain in question, individual joint limits in this initial test were normal in range. Different assistance levels or spring loads may influence overall kinematics, and the functional range for those are calculated differently and discussed in later sections.

**FIGURE 5:**
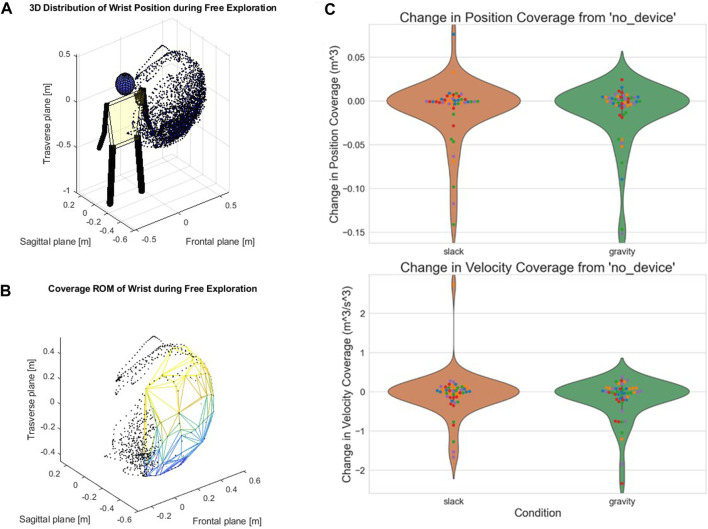
Direct Kinematic Effects in Position and Velocity Coverage. **(A)**. Example 3D point cloud distribution of wrist positions from a single movement phase (Free Exploration) of a single subject. **(B)**. Polygon formed from the convex hull algorithm. This example illustrates nearly full range of motion despite wearing the device. **(C)**. There were no kinematic changes from donning the device (orange) and from the gravity compensation condition (green), using our coverage metric. Mean change in coverage was close to 0 for the slack and gravity conditions compared to the no device condition, with mixed effects analysis revealing no significant changes between groups.

We also calculated changes in kinematics measured by position and velocity coverage metrics in the 3 device conditions. Mean changes in coverage was close to 0 for both the slack and gravity conditions compared to no device, with a mixed effects analysis revealing no significant changes between the groups (*p* > .05). This indicated that both the encumbrance of wearing the device and the experience of gravity support had minimal effects on kinematics (Figure 5). Inspecting each subject individually also revealed no significant change in kinematics.

### Wearable ExoNETs are safe to use

All participants completed the full protocol, and no injuries or safety incidents were noted. Some subjects reported that the ExoNET felt restrictive in some areas of their reachable workspace, and slight pressure in areas where the straps contacted the arm. This slight pressure was a result of straps being placed directly over EMG sensors. Every time this was reported, we adjusted strap placements for comfort.

## Discussion

Our investigation of ExoNETs on 10 unimpaired individuals yielded both promising results and identified areas for improvements. Gravity compensation with the device reduced muscular effort, specifically in the biceps, triceps, and medial deltoids, without restricting the range of motion across tasks. While muscle activities and kinematics varied across individuals and across activities, variability was accounted for in our mixed effects analysis and showed that the system reduced effort while not inhibiting kinematics.

Although this study was conducted on a healthy population, the primary goal is to adapt the ExoNET for use in individuals with upper extremity mobility impairments. This includes patients undergoing rehabilitation post-stroke. By demonstrating safety and feasibility in a healthy population, we ensure a solid foundation for subsequent trials targeting clinical populations. Future studies will specifically focus on individuals post-stroke to evaluate the effectiveness and usability of the ExoNET in enhancing the rehabilitation process.

### Muscle activity effects

Muscle activity effects were muscle-specific and related to tasks (Figure 3). As expected, tasks where the biceps were the prime mover, such as the weighted and unweighted bicep curls, resulted in the largest decreases in activity for the biceps. Similarly, tasks that targeted the deltoids, such as the shoulder raise, resulted in the largest decrease in activity at the medial deltoid. Interestingly, tasks that were predominantly performed outside of the parasagittal plane resulted in an increase in muscle activity at the medial deltoids. Deltoid muscle activity was significantly increased with the device in several tasks including shoulder circles, bicep curls, and the hair combing ADL. This is most likely due to the device’s limitation that springs partially unload and compensate less effectively for gravity when the shoulder is laterally abducted.

The cost of wearing the device is clearly identified in the sham condition which required higher effort in most activities. This was particularly evident in the medial deltoid and biceps, which naturally work against gravity. Conversely, triceps were aided by the ExoNet’s weight in activities that extended the forearm with gravity. Even so, gravity assistance in the unpowered condition led to effort reductions in all tested muscle groups: 3.6% in the biceps, 7.2% in the medial deltoid, and 4.5% in the triceps. It remains to be seen what the device’s impact might be in activities where the triceps work against gravity, such as reaching high shelves. Additionally, optimizing the ExoNet’s weight without compromising torque capabilities may lead to more significant reductions in muscular effort.

The choice of MVC for EMG normalization in this study was based on its widespread use and the ability to provide a standardized reference for comparing muscle activation levels across different tasks and individuals. MVC normalization is known for its repeatability and ease of interpretation, allowing for consistent comparisons between participants and across various activities (Bawa and Banitsas, 2022). However, MVC for some muscles may not reveal the maximal. For example, our MVC technique for medial deltoid (Figure 3) may have underestimated the maximal contraction and may be better suited with the largest contraction observed in one of the functional tasks. However, relative comparisons do not fail in showing the difference in muscle use across device conditions.

It is not known what muscle activity changes might enable individuals to regain daily activity. This study may lead to clinical application but is otherwise not a clinical study. Our percentage muscle activity gain is a relative metric that is not used clinically, and therefore there is no known *clinically meaningful improvement* level. Future clinical interventions may use similar tools to gauge improvement and relate it to function.

Future work should consider evaluating the ExoNET’s cable tension and thus torque contribution, as well as the total (ExoNET + human user) mechanical effort via inverse dynamics. This approach would help partition the ExoNET vs. user mechanical effort, inform future design choices, and add insights to the EMG data analysis presented here. Additionally, integrating normalization methods based on functional tasks could provide a more accurate assessment of muscle activation in practical scenarios and enhance the robustness of the findings.

### Kinematic effects

Kinematics were captured not using active or passive marker system but with an Xbox *Kinect* with *Body2Basics* software. Admittedly there is a tradeoff between accuracy and ease of use that may include jumps and other intermittent outlier data. However, our distribution analysis approach removes many such outliers using our *coverage* computation in exploration and other activities. Coverage tends to eliminate spurious points in the periphery of motion while doing a better job of describing multi-joint ranges of motion tendencies during function. Not all instrument errors can be removed using this technique, but the goals of this analysis were to gain an understanding of how some spurious errors caused by these instruments might be removed. It remains to be seen, however, whether a more precise measurement system might give further insights on how people can move. For example, RGB cameras have undergone extensive validation and provide improved performance compared to a Kinect camera for kinematics estimation and would be an appropriate alternative to consider in future studies (Gionfrida et al., 2022).

Velocity coverage was a secondary question, to explore whether the ExoNET might also influence speed. Velocity was not part of any explicit instruction; they were free to move in any speed that they chose. However, prior literature suggests that gravity assistance might lead to an increase in velocity (Bardi et al., 2022; Hailey et al., 2022; Moeller et al., 2022). However, we did not find any significant influence observed in either the velocity or position domains. This could be because of the tradeoff between gravity assistance and the encumbrance of a device being worn (Medrano et al., 2023). The velocity benefits of such a device remain to be seen.

### Limitations and safety considerations

While the primary goals of reduced muscle activities and no change in kinematics were met, this feasibility study revealed several limitations to prototype design. First, the ExoNET was only programmed to deliver torques in the parasagittal plane, however, most activities of daily living require motion outside of the parasagittal plane. The current design does not hinder non-sagittal motion. Prior work has demonstrated designs that can be coupled with our current design to facilitate forces during shoulder abduction (Simpson et al., 2017). Although some goals of the design were to minimize bulk and optimize donning and doffing time (minutes), several subjects still found donning time too slow and the device bulky and not aesthetically pleasing for everyday wear. Donning time was approximately measured by reviewing video recordings of the study sessions. Donning time in the first few subjects was between 5–10 min, and in the last few subjects it was between 2 and 3 min. A donning time of 2–3 min is in line with similar devices (Oliveira et al., 2019). This speed up in donning was a result of researchers having more practice donning the device on multiple subjects. This indicates that there is a learning curve that users will need to overcome to optimize donning time. Likewise, the added bulk of the device led to an increase in muscle activity, potentially impacting the effectiveness of gravity support. There are small design additions and subtractions that remain for better clinical adoption given the limited time therapists will have with patients (Celian et al., 2021). For example, additions include efficient fasteners, such as cam cleats, for decreased donning and doffing time and leveraging lighter weight materials for the ExoScales may reduce bulk which may increase the effectiveness of gravity cancellation.

We obtained very little subjective feedback from participants. A more systematic evaluation using established assessment may be better. Future studies should incorporate evaluations tests such as the System Usability Scale (SUS) or the NASA Task Load Index (NASA-TLX) to quantitatively measure user satisfaction and cognitive load during device usage. These standardized tools have been widely validated and can provide detailed feedback on various aspects of device interaction, including ease of use, perceived workload, and overall satisfaction (Hart and Staveland, 1988; Lewis, 2018). Employing these measures would ensure a more robust evaluation of the device’s usability and inform necessary design adjustments to enhance user experience. Clinically, the Intrinsic Motivation Inventory may be the best tool for understanding motivation and perceived improvement (Prange et al., 2015).

### Clinical ExoNET applications

Since the ExoNET could successfully support the arm and reduce effort across a variety of activities, it has potential in a variety of real-world settings. The passive assistance provided by the ExoNET can serve as a crucial support for post-stroke and spinal cord injured patients attempting to regain function and strength in their limbs (O’Neill et al., 2020). In industry and manufacturing, workers often face demanding physical tasks, which may lead to musculoskeletal strain over time. In such scenarios, the ExoNET has the potential to enhance worker’s performance while minimizing fatigue and injury risk. The ExoNET’s ability to be personalized to individual needs and deficits makes it a more robust option compared to devices which provide only static levels of assistance (Baldassarre et al., 2022). Indeed, the ExoNET can not only deliver gravity compensating forces, but the underlying optimization framework can approximate additional desired torques relevant to assistance and neurorehabilitation, including attractors, error augmentation, and limit push.

A safety, feasibility, and efficacy trial has been planned to study the effects of gravity compensation therapy on a post-stroke population with the ExoNET. While this device was fully safe in a healthy, unimpaired population, it remains to be seen whether the same might be true for impaired individuals with shoulder instabilities or pain during movement. Such wearable arm support devices have not been implemented extensively in clinical settings, but the ExoNET is a promising simple option to enable patients to train without adding significant limitations to the activities needed in therapy.

The ExoNET can also be utilized as a tool for resistance training and strength conditioning post-operation. This application could aid in muscle reconditioning and strength recovery in patients undergoing rehabilitation. The adjustable resistance levels provided by the ExoNET could be tailored to individual patient needs, offering a customizable and scalable solution for progressive resistance training.

## Conclusion

In summary, this investigation highlights the ExoNET’s potential in addressing gravity compensation challenges for unimpaired individuals and aiding neurorehabilitation efforts for post-stroke and spinal cord injured patients. Additionally, its utility in industrial settings could precisely reduce musculoskeletal strain in workers. The study identifies limitations including the ExoNET’s restriction to parasagittal plane motions and aesthetic concerns. Addressing these by providing any needed torque beyond the parasagittal plane, improving wearability, timing, ease-of-use, weight and torque capabilities, and expanding real-world activity could lead to broader adoption and greater muscular effort reduction.

## Data Availability

The raw data supporting the conclusions of this article will be made available by the authors, without undue reservation.
